# Biomaterials and Adipose-Derived Mesenchymal Stem Cells for Regenerative Medicine: A Systematic Review

**DOI:** 10.3390/ma14164641

**Published:** 2021-08-18

**Authors:** Vivian Alonso-Goulart, Loyna Nobile Carvalho, Ana Leticia Galante Marinho, Bianca Lourenço de Oliveira Souza, Gabriela de Aquino Pinto Palis, Henrique Guerra Drumond Lage, Isabela Lemos de Lima, Laura Duarte Guimarães, Lucas Correia Peres, Márcia Marques Silveira, Gilberto Henrique Nogueira Lages Lopes, Lorraine Braga Ferreira, Letícia de Souza Castro-Filice

**Affiliations:** 1 Laboratory of Nanobiotechnology, Institute of Biotechnology (IBTEC), Federal University of Uberlândia, Uberlândia 38400-902, Brazil; loyna.nobile@gmail.com (L.N.C.); analeticia99@hotmail.com (A.L.G.M.); bianca121oliveira@gmail.com (B.L.d.O.S.); gabriela.palis05@gmail.com (G.d.A.P.P.); henriquedlage@gmail.com (H.G.D.L.); Isabela.lemosl@hotmail.com (I.L.d.L.); lauraduarte2915@gmail.com (L.D.G.); perescorreialucas@gmail.com (L.C.P.); marciamarquessilveira@gmail.com (M.M.S.); lorraine.braga@gmail.com (L.B.F.); 2 Faculty of Medicine, Federal University of Uberlândia, Uberlândia 38400-902, Brazil; ghnllopes@gmail.com (G.H.N.L.L.); leticiafilice@ufu.br (L.d.S.C.-F.)

**Keywords:** biomaterial, stem cells, tissue engineering

## Abstract

The use of biological templates for the suitable growth of adipose-derived mesenchymal stem cells (AD-MSC) and “neo-tissue” construction has exponentially increased over the last years. The bioengineered scaffolds still have a prominent and biocompatible framework playing a role in tissue regeneration. In order to supply AD-MSCs, biomaterials, as the stem cell niche, are more often supplemented by or stimulate molecular signals that allow differentiation events into several strains, besides their secretion of cytokines and effects of immunomodulation. This systematic review aims to highlight the details of the integration of several types of biomaterials used in association with AD-MSCs, collecting notorious and basic data of in vitro and in vivo assays, taking into account the relevance of the interference of the cell lineage origin and handling cell line protocols for both the replacement and repairing of damaged tissues or organs in clinical application. Our group analyzed the quality and results of the 98 articles selected from PubMed, Scopus and Web of Science. A total of 97% of the articles retrieved demonstrated the potential in clinical applications. The synthetic polymers were the most used biomaterials associated with AD-MSCs and almost half of the selected articles were applied on bone regeneration.

## 1. Introduction

### 1.1. Biomaterial: The Biological Generation Template

Biomaterials, natural or synthetic, composites, ceramics and metals alive or lifeless, are being defined as materials that interact with biological systems [1]. The fully interactive, biocompatible, biodegradable and non-cytotoxic biological system framework is still widely used in regenerative medicine to assist in treatments of wounds and diseases, also enabling the creation of substitutes for medical devices. Biomaterials are biosynthesized in micro/nanometric terms, with basic structural units, grains, particles, fibers or other constituent components, bigger/smaller than 200 nm, also in all sizes and shapes [2]. The affluent diversity of surface and characteristics defines all types of functions, designed to interact with cellular and molecular events well, interfering with their biological activities with no toxicity [3]. The wide variety of clinical applications favor the design of unique techniques, such as tissue engineering, drug delivery, bioimaging, gene therapy and 3D bioprinting [4]. In addition, the combination of different techniques to prepare compartmented scaffolds is a promising path to reveal not only sophistication in the translational studies, but a challenge to approach accordingly the volume of numerous scaffold designs and application methods, bringing reliable sources for repairing the tissue or organ.

Actually, supporting cells during the formation of new tissues is a quite amazing and promising ability of biomaterials; in addition, the fabricated scaffolds outweigh their advantages in supporting cell adhesion, proliferation and growth, by controlling the biochemical and mechanical properties of the microenvironment for successful cell delivery [5]. By decreasing the size of the material to the nanoscale with nanotechnology, the surface area is dramatically increased and the correlation between surface area and volume can create superior physicochemical properties [6].

### 1.2. Stem Cells: Origin, Design and Differentiation

One of the first records about stem cells came with Danchakoff V. (1916), who, in particular, took advantage of the differentiation of cells as a criterion for cell identification. Some time later, a model for differentiation of hematopoietic stem cells was shown by Wolf and Trentin (1970). “Mesenchymal Stem Cells” was shown a few years after by Caplan (1991). In 2019, the International Society for Cellular Therapy (ISCT) published a short communication about a new position statement on nomenclature and the affirmation was for the use of MSC for mesenchymal stromal cell [7].

Stem cells are described as cells with the capacity of differentiating into one or more types of cells and those are commonly called specialized cells. According to the origin of the cells, they can be classified as either embryonic or adult stem cells. Embryonic stem cells (ESCs), despite being pluripotent and able to be differentiated into any type of cells, face problems regarding their use because of ethical issues related to human embryos, which are used for obtaining the cells [8,9]. In addition, some studies have shown that samples of ESCs can have possible tumor-promoting cells, as demonstrated [10]. Adult stem cells have a limited growth potential, if compared with embryonic stem cells, but are most likely to fit ethical regulations for use in research. Mesenchymal stem cells (MSCs) are one of the groups of adult stem cells and they have important characteristics, such as the capability of differentiation into adipogenic, chondrogenic, osteogenic and even neurogenic cells. These cells can also show immunomodulatory properties, as well as secrete bioactive molecules, making them valuable for therapeutics in degenerative and autoimmune diseases [9,11]. MSCs are classified according to criteria established by the International Society for Cellular Therapy (ISCT) and some of the requirements to be met are adherence and fibroblastoid morphology, as well as surface markers, such as CD105, CD73 and CD45, for example. In addition, they must be able to differentiate into osteoblast, chondrocyte or adipocyte cell lineages in vitro [8,11].

Bone marrow (BM) is a widely known and used source of MSC, but it can be obtained from several adult tissues. For instance, adipose tissue (AT) is another great source of MSC and also dental tissues, extra-embryonic tissues (such as amniotic fluid, amniotic membrane, fetal membrane and placenta), endometrium and Wharton’s jelly [12,13]. The procedure to obtain stem cells from BM is very invasive and painful. On the other hand, the harvesting process of adipose-derived stem cells (AD-MSCs) is fairly simple, considering that liposuction and bichectomy surgery waste material contain these cells [13]. Buccal fat pad (BFP), which can be obtained from bichectomy surgery, are cells phenotypically similar to AD-MSCs obtained from liposuction surgery [14]. Moreover, this source has drawn attention for being very accessible. It is also known that the concentration of AD-MSCs in adipose tissue is almost five hundred times the concentration of BM-MSC in bone marrow [15]. In conclusion, AD-MSC and BFP are promising sources of MSC, due to their easy harvest and for being a source of stem cells of great value [13,14]. In addition, they have prominent implications in tissue regeneration due to their ability to differentiate into multiple lineages and to secrete various cytokines, as well as having immunomodulatory effects [16].

### 1.3. Our Efforts

**Figure 1:**
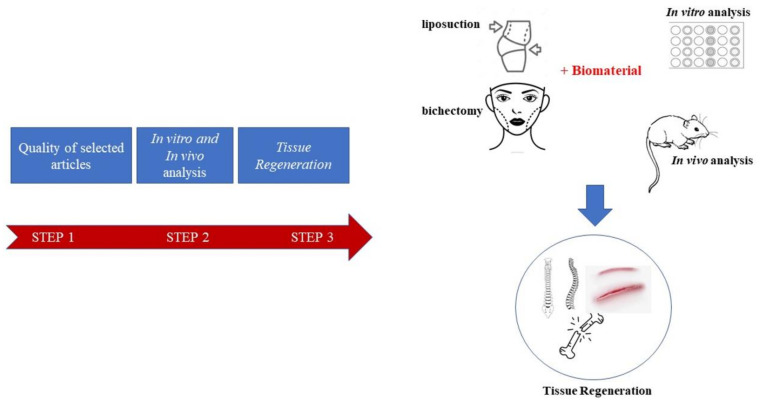
Schematic flow diagram of the review’s objectives.

This review is placed on the quality of selected articles, in vitro and in vivo studies of AD-MSCs associated with different biomaterials for different clinical applications (Figure 1). We also sought to identify the possible correlation between the current methods of their isolation, culture and characterization, taking into account whether the biomaterials exerted a positive effect on histological outcomes in the in vivo application models, with emphasis on performing the homogeneity in the field, publication bias and study quality scores, which may be another source of bias for standardization of outcome measures and improved study reporting. Furthermore, the application in regenerative medicine is summarized and, lastly, the future clinical applications of AD-MSCs are discussed.

## 2. Materials and Methods

### 2.1. Literature Search in PubMed, Scopus and Web of Science (Flow Diagram)

This systematic review was performed to address the following theme: origin, nature and applications of biomaterials and AD-MSCs for regenerative medicine.

A systematic review of literature for biomaterials and human adipose-derived mesenchymal stem cells was performed and reported according to the PRISMA criteria (Preferred Reporting Items for Systematic Reviews and MetaAnalyses) [17]. Searches were carried out on 10 April 2020 in Pubmed, Web of Science and Scopus using the following keywords and subject terms: “Buccal fat stem cell”, “Buccal fat pad”, “Buccal fat stem cell and regeneration”, “Buccal fat pad and regeneration”, “Buccal fat stem cell and nanomaterial”, “Buccal fat pad and nanomaterial”, “Buccal fat stem cell and biomaterial”, “Buccal fat pad and biomaterial”, “Buccal fat stem cell and regeneration and nanomaterial”, “Buccal fat pad and regeneration and nanomaterial”, “Buccal fat stem cell and regeneration and biomaterial”, “Buccal fat pad and regeneration and biomaterial”, “Buccal fat stem cell and adipose-derived stem cell”, “Adipose-derived stem cell and regeneration and nanomaterial” and “Adipose-derived stem cell and regeneration and biomaterial”.

According to the eligibility criteria, the authors selected the studies independently in two stages: evaluating the title and summary and, subsequently, reading the full text. Disagreements were resolved with consensus.

In the first stage, the studies were screened by title and abstract using the following inclusion criteria: (a) research articles, (b) complete articles, (c) impact factor above 2, (d) published between 2010 and 2020, (d) English language, (e) human cells and (f) primary culture of adipose tissue stem cells (from liposuction or bichectomy). The studies that were excluded were those whose articles did not set the origin of tissue to obtain adipose-derived mesenchymal stem cells, had an impact factor less than 2, used only non-human stem cells, were review article/systematic review/clinical trial/case report/meta-analysis/books, were in languages other than English, were published outside the 2010–2020 period and did not use the primary culture of adipose-derived mesenchymal stem cells. The repeated articles within the individual databases, as well as among different databases, were manually deleted.

In the second stage, the authors screened the studies for eligibility by reading the full text. The exclusion criteria included collecting adipose-derived stem cells from unhealthy patients, adipose-derived stem cells obtained from commercial lineage, studies that evaluated only the biomaterial, sample collection by other procedures that did not fall in the liposuction and bichectomy categories, studies that used only non-human stem cells (that was not specified in the title and summary), studies that did not set the origin of the tissue to obtain adipose-derived mesenchymal stem cells (this lack of information was not explicit in the title and summary) and studies without application in regenerative medicine. We did not exclude papers that, in addition to liposuction and bichectomy, cited other sources nor articles that declared conflicts of interests.

### 2.2. Questions for the Quality Assessment of Retrieved Articles

The following questions constituted the tool used to assess the quality and bias of the selected studies: (1) Was there a clear statement of the aims of the research? (2) Was the research design appropriate to address the aims of the research? (3) Was the execution of the methodologies described in sufficient detail to permit replication of the experiments? (4) Did the study provide a clear definition of what was considered to be a positive and negative control? (5) If necessary, have ethical issues been taken into consideration? (6) Was the data analysis sufficiently rigorous? (7) Was the study free of commercial funding? (8) Was the characterization of MSC conducted according to The International Society for Cell & Gene Therapy (ISCT^®^) criteria? (9) Were the MSCs used between the 2nd and 5th passages in experiments? (10) Did the MSCs demonstrate the potential for osteogenic, chondrogenic or adipogenic differentiation in vitro? (11) Did the research results show the potential of MSC in clinical application?

The first seven questions were obtained from the Cochrane Handbook for Systematic Reviews [18]. The other questions (8, 9, 10 and 11) were included for the analysis of the stem cell characterization that defines human MSC (mesenchymal stromal cells). The minimum criteria were defined by the Committee of the International Society for Cellular Therapy (ISCT) and aimed to decrease contrasting study outcomes [19].

## 3. Results

### 3.1. General Findings (Flow Diagram Results)

**Figure 2:**
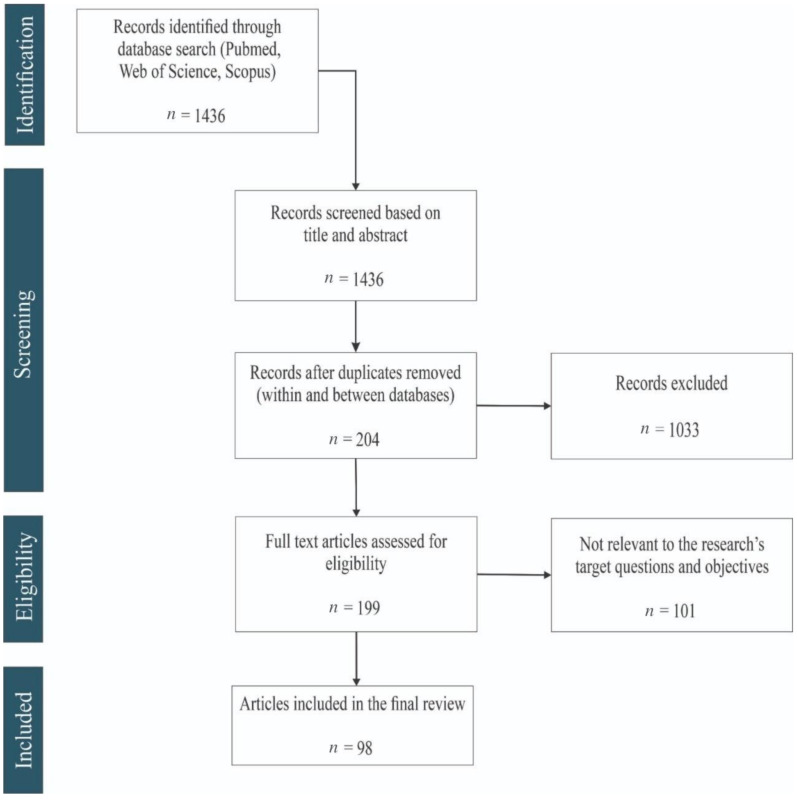
PRISMA chart showing the screening process for the selection of eligible studies.

The electronic search process in the databases output 1436 studies. Among them, 98 articles published in 2010–2020 met all inclusion criteria (Figure 2).

### 3.2. Quality of the Selected Articles

**Figure 3:**
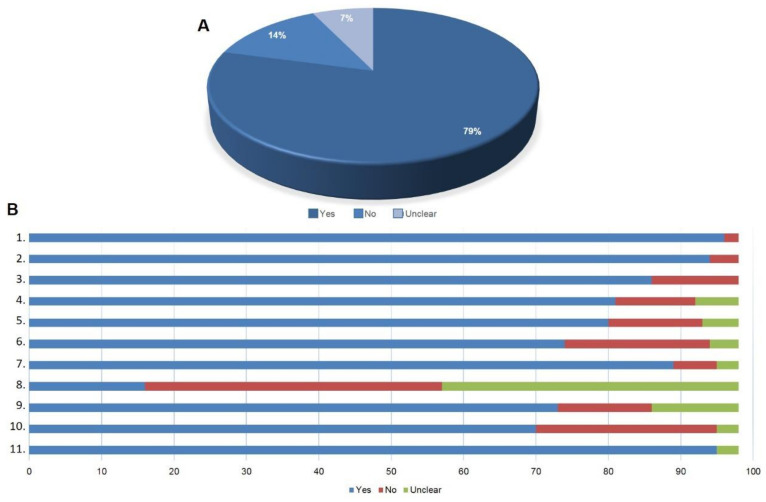
Analysis of the quality of the selected articles. (**A**) Overall retrieved articles. (**B**) Quality of the selected studies according to the eleven pre-defined assessment questions: 1. Was there a clear statement of the aims of the research? 2. Was the research design appropriate to address the aims of the research? 3. Was the execution of the methodologies described in sufficient detail to permit replication of the experiments? 4. Did the study provide a clear definition of what was considered to be a positive and negative control? 5. Have ethical issues been taken into consideration? 6. Was the data analysis sufficiently rigorous? 7. Was the study free of commercial funding? 8. Has the characterization of MSC been done according to ISCT criteria? 9. Were MSC used from the 2nd to the 5th passage in experiments? 10. Did MSCs demonstrate the potential for osteogenic, chondrogenic or adipogenic differentiation? 11. Did the research results show the potential of MSC in clinical applications? Yes (blue), No (red) and Unclear (green).

More than three quarters of the questions about the quality of the selected articles were answered positively, showing the good quality of the retrieved articles. Only 7% of the questions got an unclear answer and 14% a negative answer to the established criteria (Figure 3A).

Only two selected articles did not state clearly the aims of the research, but most of the articles (95.9%) showed an appropriate experimental design. Most of the articles described the methodologies in sufficient detail to make room for the replication of the experiments. Less than 12% of the studies did not provide a clear definition for positive and negative controls. When biological samples were used, more than 80% considered ethical issues. We considered that around 75% of the articles reported sufficiently rigorous analyses of their data and only 20% of the results sections were poorly described.

However, more than 6.1% of the articles were funded by companies being susceptible to potential conflict of interests. The majority of MSC used in the retrieved articles did not inform that the characterization of the cells had been performed according to ISCT (International Society of Cellular Therapy). More than 74% of the selected articles used the cells from 2nd and 5th passages in experiments; 71.4% of the retrieved articles demonstrated the potential of cell differentiation and almost 97% showed the potential in clinical application (Figure 3B).

### 3.3. Publication Overview between 2010 and 2020

**Figure 4:**
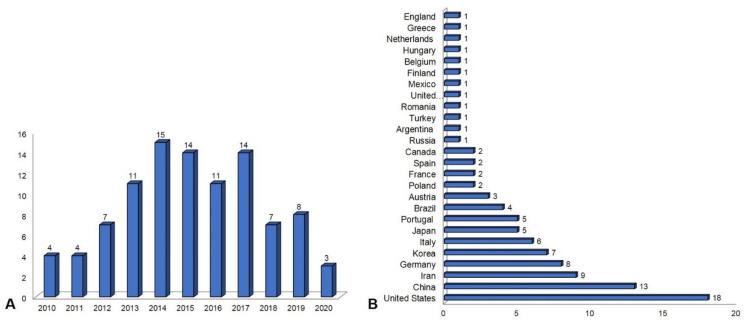
Overview of the numbers of publications about biomaterials and AD-MSCs for regenerative medicine worldwide. (**A**) Numbers of publications between 2010 and 2020. (**B**) Numbers of publication per country.

The search was limited to the papers with dates of publication between 2010 and 2020. The years 2014, 2015 and 2017 had the highest concentration of paper publications, as it is shown in Figure 4A. During these years, there were publications in twenty-five different countries and highlights with the greatest number of submissions in the United States and China (Figure 4B).

### 3.4. Biomaterial Used with AD-MSCs for Regenerative Medicine

In this review, information on the use of biomaterials associated with mesenchymal stem cells was extracted from the selected articles and arranged according to a previous study by Pires et al., 2015. The classification based on composition was divided into categories, namely, metals, synthetic polymers, natural polymers, ceramics, composites and their blends [20].

Metals include materials such as titanium disks, gold nanoparticles and cobalt, which demonstrated a high mechanical performance and high resistance to fatigue and fractures. Ceramics have a high chemical compatibility with the physiological environment and with rigid tissues, such as teeth and bones; alumina, zirconia, calcium phosphate and bioglass are examples of ceramics. Polymers are divided into two categories—synthetic and natural. The synthetic ones, hydrophilic or hydrophobic, such as polyesters, polyamides and polyethylenes, (PTFE, PLA, PVP, PVA and hydrogels) ensure a high variety of functional properties, allowing the control of the mechanical properties. Natural polymers, chitosan, alginate and hyaluronic acid, carbohydrates, glycolsaminoglycons and agaroses, are highly affordable and obtained from renewable sources [20]. The classification “Others” grouped biomaterials that did not fit into the categories mentioned above.

**Figure 5:**
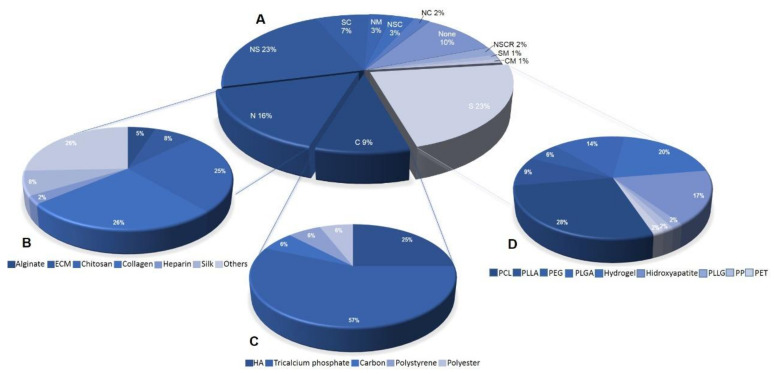
(**A**) Types of biomaterial associated with AD-MSC for regenerative medicine. S (synthetic polymers), N (natural polymers), C (composite), NS (natural and synthetic polymers), SC (synthetic polymers and composite), NM (natural polymers and metals), NC (natural polymers and composite), NSC (natural and synthetic polymers and composite), NSCR (natural and synthetic polymers and ceramics), SM (synthetic polymers and metal), CM (composite and metal) and None (not used or not reported). (**B**) Types of natural polymers (alginate, extracellular matrix, chitosan, collagen, heparin, silk and others). (**C**) Types of composites (HA, hyaluronic acid, tricalcium phosphate, carbon, polystyrene and polyester). (**D**) Types of synthetic polymers (PCL, PLLA, PEG, PLGA, hydrogel, hydroxyapatite, PLLG, PP and PET).

Within the analyses conducted in this review, we were able to observe that most articles make use of synthetic polymers, corresponding to 21% of the total quantity. Sequentially, the most used are natural polymers, with 15%. Composites comprised 9% of the number of biomaterials mentioned and their blends 39%. Those that did not use any type of biomaterial accounted for 9% and, finally, the classification “Others” represented 7% of the biomaterials (Figure 5A). Among the most reported natural polymers are collagen (26%) and chitosan (25%) (Figure 5B). Tricalcium phosphate was the most used composite biomaterial (57%), followed by hyaluronic acid (25%) (Figure 5C). The most used synthetic polymers were PCL (28%) and hydrogel (20%), followed by hydroxyapatite, PLGA and PLLA (Figure 5D).

**Figure 6:**
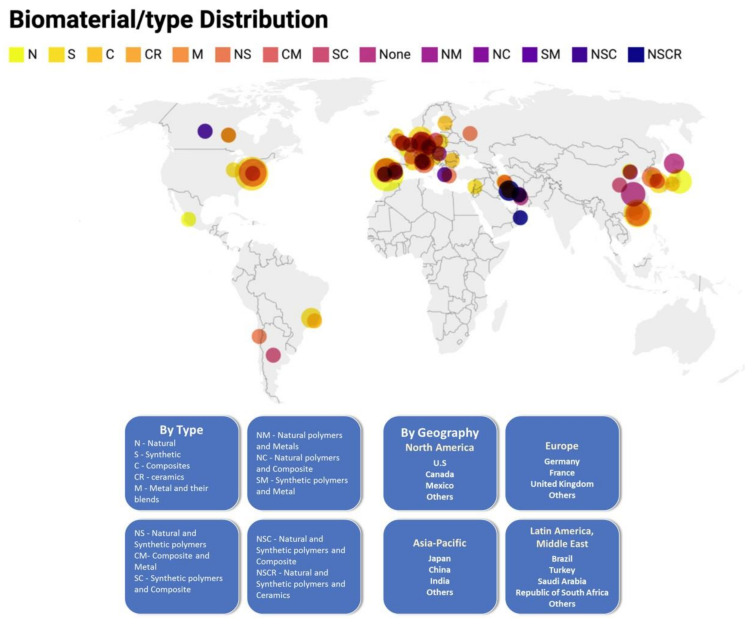
A bivariate dot distribution density map showing the relative type and concentration of the biomaterial used in each country (N, natural; S, synthetic; C, composites; CR, ceramics; M, metal and their blends—NS, natural and synthetic polymers; CM, composite and metal; SC, synthetic polymers and composite; NM, natural polymers and metals; NC, natural polymers and composite; SM, synthetic polymers and metal; NSC, natural and synthetic polymers and composite; NSCR, natural and synthetic polymers and ceramics). Biomaterial division map modified from Ratner B. et al., 2012, Biomaterial Science [21].

In recent years, the pre-clinical combination of biomaterials with stem cells has shown increased interest in the development of biomaterial-based therapies to promote tissue repair and functional recovery all over the world. Here, the authors found different blending strategies utilizing biomaterials as structural support for tissue regeneration or as delivery vehicles for AD-MSC. While a range of biomaterials has been tested, currently no overview is available to show the distribution of each material type according to country/region (Figure 6). The report analyzes the biomaterials used in addition to AD-MSC based on type, namely, metallic, polymeric, ceramic and natural types, as well as their blends. Metallic biomaterials are further segmented into stainless steel, titanium and titanium alloys, cobalt–chrome alloys, gold and silver. Polymeric biomaterials are further divided into polymethyl methacrylate (PMMA), polyethylene, polyester, polyvinylchloride, silicone rubber, nylon and polyetheretherketone. Ceramic composite biomaterials are further segmented into calcium phosphate, zirconia, aluminum oxide, calcium sulfate, carbon and glass, hydroxyapatite, polycarbonate (PC), polyethylene (PE), polypropylene (PP), polystyrene (PS), polytetrafluoroethylene (PTFE) and PMMA. Natural biomaterials included hyaluronic acid, collagen and gelatin, fibrin, cellulose, chitin, alginates and silk. The studies showed the clinical application into wound healing, neurological disorders, drug-delivery systems, bone disorders, cartilage growth and soft tissue reconstruction.

A homogeneous distribution was found regarding the use of pure biomaterials and doubly blended as natural + synthetic with great representation in North America, Europe, Asia-Pacific, Latin America and the Middle East. Metals were less homogeneous, having their representation only as blends in two European countries, Asia-Pacific and Iran. Another important highlight was the low representation of triple blends, such as natural+composite+ceramic/synthetic, appearing in only three regions, including Canada, Iran and Oman; the double blends were very representative in North America, Europe, Asia-Pacific, Latin America and the Middle East (Figure 6).

### 3.5. Adipose Tissue Resources for AD-MSCs Isolation, Culture and Characterization

**Figure 7:**
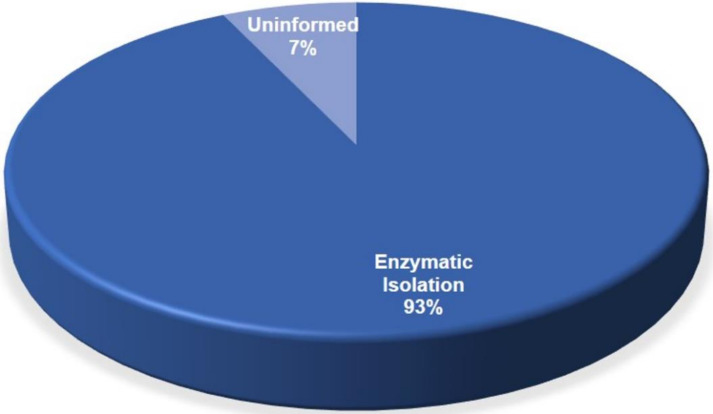
Isolation methodology of AD-MSC.

Two areas of the adipose tissue resources were chosen, liposuction and bichectomy (buccal fat pad), with 86% and 14%, respectively. The isolation methodology of the adipose mesenchymal stem cells in all articles have been analyzed; as a result, in 93% of the total, most parts of the cells have been isolated enzymatically. A total of seven articles did not specify the methodology that was used for isolation and those were classified as an “uninformed” category (Figure 7).

The isolation methodology of liposuction and bichectomy did not show differences. Both used the enzymatic methodology with the collagenase type I in the concentration of 0.075–0.1%.

**Figure 8:**
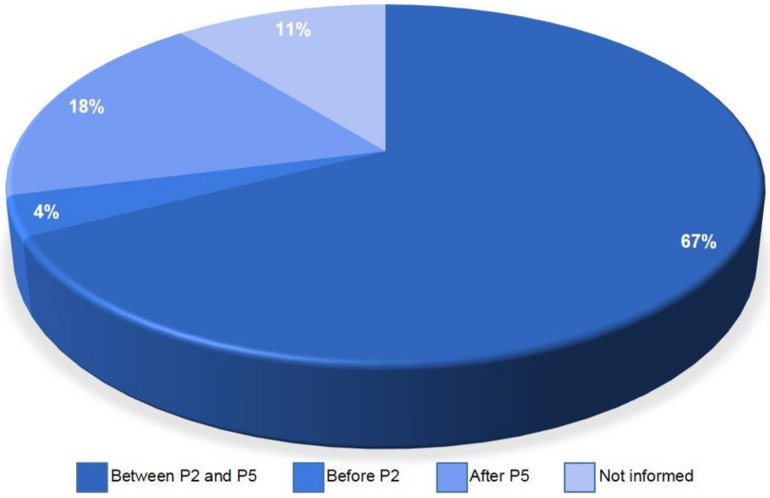
Number of cell culture passages for the experiments using AD-MSC, divided into 4 sections: not informed, after 5th passage (P5), before 2nd passage (P2) and between P2 and P5.

Every time the population of cells is split into another culture flask, a new subculture is created [22]. Some articles used more than one passage to make their research, with the total passages being from 1 to 17 (~89.22%); these were then categorized into three classes, namely, cell culture between 2nd and 5th passages (~67%), after 5th passage (~18%) and before 2nd passage (4%). In addition, approximately 11% of the articles did not provide any information about the cell culture passage (Figure 8). 

**Figure 9:**
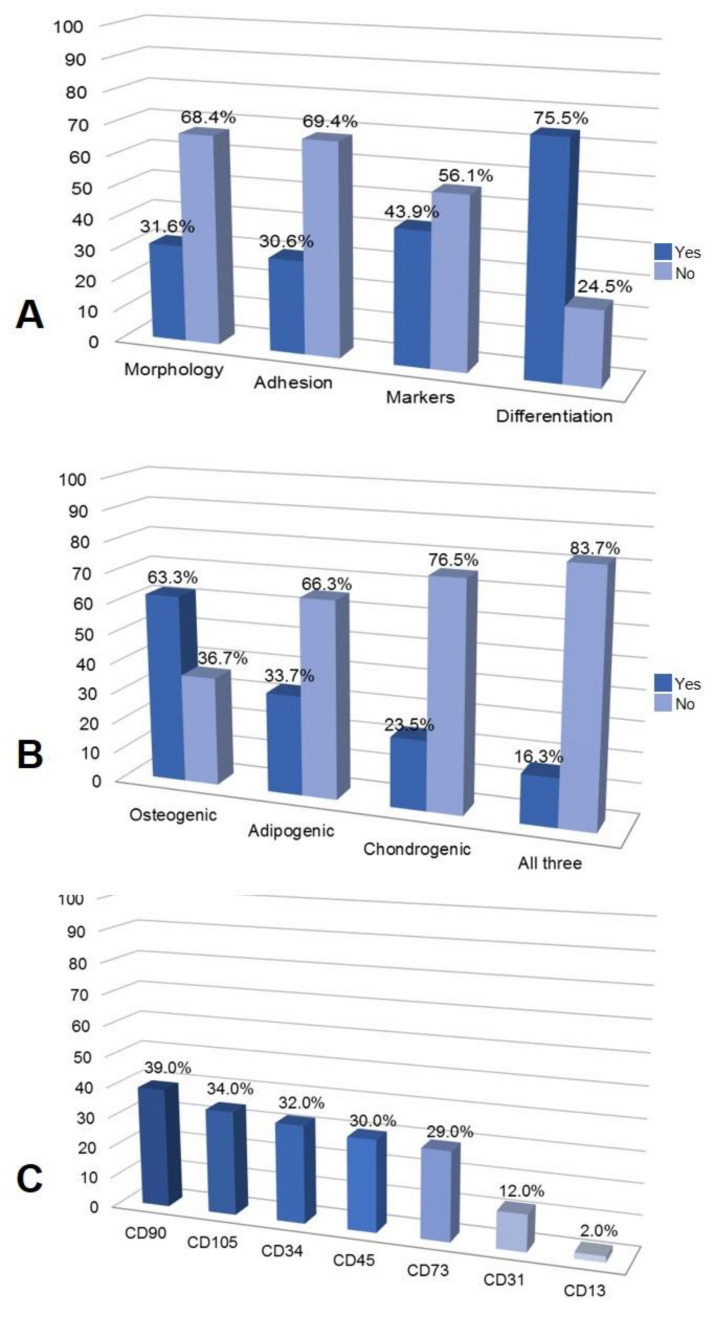
AD-MSCs characterization. (**A**) % of selected articles that used the International Society of Cellular Therapy criteria for characterization—morphology, adhesion, markers and differentiation. (**B**) Osteogenic, adipogenic, chondrogenic differentiations or all differentiations analyzed in the selected articles. (**C**) Markers analyzed in the selected articles.

As for the characterization of stem cells, a good part of the 98 articles used the criteria already described in the literature as standards. As shown in Figure 9A, around 68.4% of the articles did not mention fibroblastoid morphology as a parameter for characterizing the cells. A large number (69.4%) did not mention adhesion to plastic. The chondrogenic, osteogenic and adipogenic differentiation trials were neglected by 75.5% of the articles, the majority of which were already focused on differentiating the study target (Figure 9B).The characterization by surface markers analyzed by flow cytometry was mentioned by slightly less than half (43.9%) of the articles in question; it is important to assess that an important part of these studies did not do so, raising the hypothesis that it may be something already intrinsic to the use of this type of cells. Among these articles, the present markers CD90 (39%), CD105(34%) and CD73 (29%) and the absent ones CD34 (32%), CD45 (30%), CD31 (12%) and CD13 (2%) were cited (Figure 9C).

### 3.6. In Vitro and In Vivo Assays

The 98 selected papers were categorized regarding the types of in vitro experiments described on the paper and grouped within 11 categories as outlined in Table 1: electronic microscopy, optical microscopy, ELISA, immunohistochemistry, immunocytochemistry, spectrometry, genetic testing, metabolic assays, viability and cytotoxicity, flow cytometry and “Others”. Moreover, three articles did not describe any type of microscopy used and there was a noteworthy variety of testing types among the articles.

A total of 95 (96.93%) articles presented some sort of microscopy, with 93.88% (92 articles) having utilized optical microscopy techniques and 52.04% (51 articles) making use of electronic microscopy, most commonly scanning electron microscopy (SEM, in 44 articles). Regarding immunochemical assays, a total of 41 articles (41.84%) described either immunohistochemistry or immunocytochemistry testing—with only 1 article having both. Additionally, there was a larger overlap between ELISA testing and the use of other specific spectrometry-based assays (11 articles), than with other immunochemical assays (5 articles). Regarding genetic evaluation (62.24%), the majority (56) of the articles made use of real-time quantitative polymerase chain reaction (RT-PCR). The five remaining articles either only used quinacrine (QFQ) chromosomal analysis, fluorescence-staining-based quantitation assays or, in the case of a single article, the testing was not described in the body of the work.

There was significant variety among the methods employed for evaluating metabolic assays (50%) and viability and cytotoxicity (32.66%). The most used for metabolic assays was the alkaline phosphatase (ALP) test, in 32 articles. Viability/cytotoxicity analysis of intracellular tetrazolium salts was used in 14 articles with full methyltetrazolium (MTT) assays and 4 articles with Methyl tetrazolium salt (MTS); the LIVE/DEAD fluorescence-based assay was present in 18 articles. Lastly, the most common assays of the other category (44.90%) were mechanical testing of differentiated tissue (7) and immunoblotting or Western blot (6)**.**

**Figure 10:**
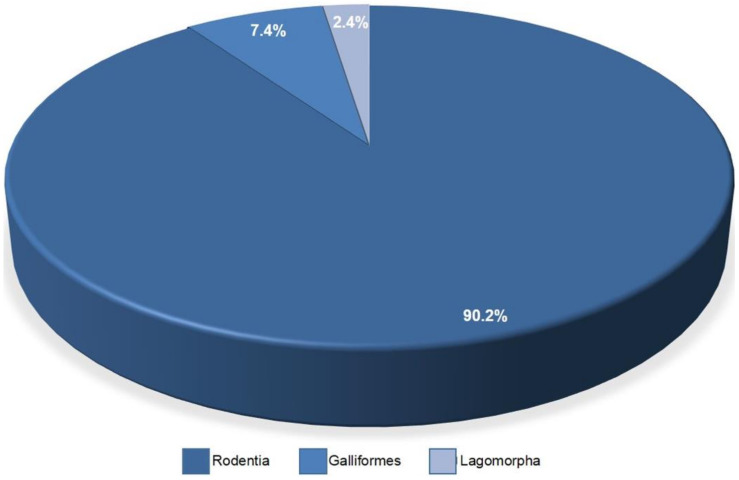
Types of animals used in the in vivo assays.

In vivo assays were performed in 41.8% of the articles. Different types of animals were found in the search, but most of them (~90.2%) were from Rodentia models, 7.4% were from chicken chorioallantoic membrane (CAM) in the order Galliformes and only one article (2.4%) used Lagomorpha as model organisms (Figure 10).

### 3.7. Regenerative Medicine Applications

**Figure 11:**
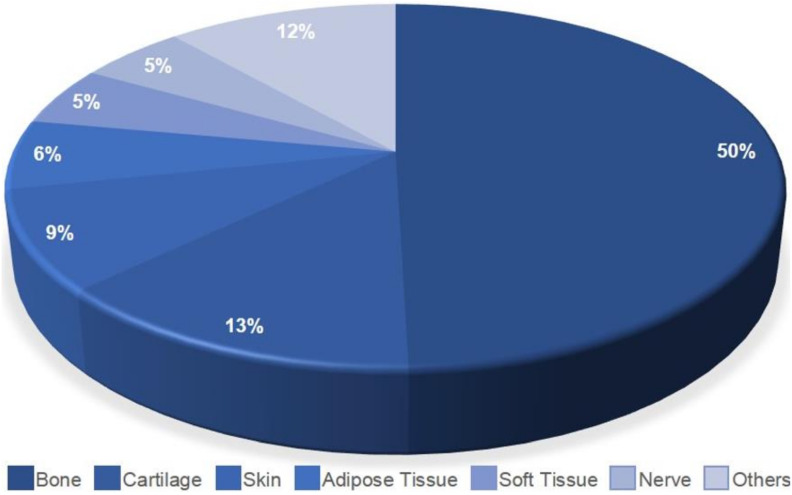
Types of applications in AD-MSCs for regenerative medicine.

Half the articles selected in this work focused on bone regeneration. As for the remaining articles analyzed, 13% were for cartilage regeneration, 9% for skin, 6% for adipose tissue, 5% for soft tissue and nerve and 12% focused on various areas, such as neurons, myocardial regeneration and muscle (Figure 11).

## 4. Discussion

### 4.1. The United States and China Published More Papers on AD-MSC and Nanomaterials Than Other Research Centers

AD-MSCs researchers increased from 2010 to 2014, but started to decrease between 2017 and today. The 98 retrieved papers were produced by research teams from 25 countries, with USA and China being the countries that published the largest number of articles in this field, probably because these countries have the best financial, structural and human resources conditions to carry out these surveys. Probably, the AD-MSCs researchers decreased after 2017 because of the increase in the use of Induced Pluripotent Stem cells (IPSC) for regenerative medicine.

### 4.2. Biomaterials Used for Applications in Regenerative Medicine

Advances in biomaterial sciences have added extremely valuable tools to manipulate cell behavior for applications in regenerative medicine [23]. They have been designed to act as carriers to deliver cells to desirable regions for local tissue regeneration and also to stimulate host cell recruitment, homing and differentiation [24]. Biomaterial-based techniques can better mimic native tissue and organ structure and influence the biocompatibility with the host organism [25]. An ideal biomaterial must be able to increase the survival rate of MSCs, appropriate their cell function after transplantation and stimulate functional tissue growth in situ, while promoting its own degeneration with the completion of treatment [26].

Types of biomaterials can show different biochemical and biomechanical aspects that influence the proliferation, migration and differentiation of AD-MSCs.

Among the articles analyzed, the most used biomaterial was polycaprolactone (PCL), comprising 43.7% of appearances in the category of synthetic polymers, as stated by Hung, BP et al., 2016 [27]. Using PCL as the “binder” to improve tissue regeneration is extremely valid due to the readily printable, minimal flow away from the target print location, attributable to high viscosity, besides the fact that it is not cytotoxic. Similar to PCL, another synthetic polymer, poly(l-lactide-co-ε-caprolactone) (PLCL) was manufactured as a scaffold in a study by Jeong SI, et al., 2012 [28]. Using a scCO2–HFIP co-solvent system, it was able to deliver chemical and mechanical signals for a certain period of time for differentiation, testifying that PLCL would be useful as a functional scaffold for cartilage tissue engineering [27]. The pore size of PCL was evaluated on ASC osteogenesis. PCL scaffolds with stiff pore walls led to an enhanced osteogenic response as verified by histology, biochemical analysis and mechanical tests according to Huri et al., 2014 [29]. Cardoso et al., (2017), investigated the oleic acid (OA) on PCL potential as a bone induction factor and PCL/OA scaffolds exhibited porosity and wetting property sufficient to support cell proliferation and adhesion with a positive effect on ADSC cell differentiation [30]. Zhang et al., 2017, used adipic dihydrazide (ADH) to cross-link PLGA to form a hydrogel, followed by freeze-drying to achieve a porous structure that performed as an elastic material. Such material possessed high swelling capacity, inducing AD-MSCs to form multicellular spheroids, which demonstrates the potential of the present strategy in vascularized adipose tissue engineering [31].

The rates of applications of natural polymers as biomaterials were also notable. Within that use, the biomaterial that stood out as the most frequently used was chitosan, which has been reported to have antimicrobial and wound-healing properties. Denost et al., 2015, showed the capacity of chitosan scaffold to support the regeneration of smooth muscle layers in vivo, allowing better wound healing, when compared with different kinds of biomaterials, due to a more effective control of inflammatory activity and a full regeneration of the colonic wall, including the smooth muscle cell layer [32]. Silk-based anisotropical 3D biotextiles have a great capacity to fold and to be easily adjusted and shaped to the bone defect, facilitating surgical intervention. Silk fibroin (SF) yarns were processed into weft-knitted fabrics spaced by a monofilament of polyethylene terephthalate (PET) presenting lower porosity and mean pore size at the surface, that might induce a higher stiffness of the structures in the hydrated state. According to Ribeiro et al., 2017, and Bellas et al., 2013, this scaffold presented suitable porosity and mechanical properties for bone applications and also showed a lipoaspirate combined with a silk protein matrix in a subcutaneous rat model, allowing for tissue ingrowth while remaining mechanically robust during 18 months [33,34]. The silk sponge acted as a template for dynamic tissue regeneration even during this remodeling process [34]. Regarding the ceramics scaffolds, using them as a coated layer can cover the surface without closing the macropores, improving both mechanical and biological properties of the scaffold. Hydroxyapatite (HA) and tricalcium phosphate (TCP) were the most outstanding ceramics, according to the articles analyzed in this review. As believed by Jun-Beom Park et al., 2013, hydroxyapatite-coated surfaces demonstrated the highest alkaline phosphatase and osteocalcin expression. Using HA as coatings was exalted based on the fact that this biomaterial can provide the host’s bone cells with a number of mineral elements involved in the metabolism of osteoblasts, accelerating osseointegration [35]. Bastami et al., 2017, reported that tricalcium phosphate displayed osteogenic property, phase stability and strong bond formation with the host bone tissue; in addition to having a Ca/P ratio similar to that of natural bone tissue, TCP also showed progressive biodegradability accompanied by the simultaneous formation of normal bone structure in vivo [36].

Using ECM as a biomaterial has not been widely reported in the articles, but it has a promising potential for applications in tissue engineering and regenerative medicine. A human ECM-derived scaffold was developed in a study by Choi et al., 2010, to chart a new path to clinical challenges in patients suffering from soft-tissue defects or needing soft-tissue augmentation. They claimed that this technique can find broad clinical utility in soft-tissue engineering, not only for aesthetic plastic surgery, but also for regeneration of tissues lost as a result of extensive deep burns, tumor resection and hereditary and congenital defects [37].

Gelatin hydrogels have a wide range of ramifications with different specificities and benefits. To illustrate, Chattopadhyay et al., 2019, developed a tendon hydrogel capable of improving the repair strength after a tendon injury. This tendon hydrogel is biocompatible, crosslinks at human body temperature and supports the proliferation of AD-MSCs [38]. In a study by Eke et al., 2017, Methacrylated Gelatin (GelMA) hydrogels loaded with ADSC showed great angiogenic and proliferative essential properties for the promotion of angiogenesis to wound healing, helping to improve the survival of tissue-engineered skins [39].

Metals were the least cited biomaterials in the articles evaluated. However, they demonstrate a high mechanical performance and high resistance to fatigue and fractures and, according to Tátrai et al., 2012, the combination of metals such as titanium with mesenchymal stem cells can promote osseointegration and also stimulate bone regeneration. An accelerated adhesion between metal and adjacent bone was noticed when the MSCs were seeded on titanium alloy plates [40]. Besides titanium, a study by Razavi et al., 2019, attested that gold nanoparticles (AuNPs) are able to enhance the proliferation and differentiation of stem cells. AuNPs also have excellent biocompatibility, antimicrobial activity and a high mechanical strength [41].

For the hybrid biomaterials, gold nanoparticles encapsulated in chitosan nanoparticles had no cytotoxic effect on AD-MSCs viability. In addition, the delivery of NGF and AuNPs through chitosan nanoparticles increased the differentiation of h-ADSCs into Schwann-like cells and myelinating capacity in vitro [41]. Another work showed that the AD-MSCs isolated from buccal fat pad were seeded on the modification of titanium surfaces by sand-lasted/acid-etching and hydroxyapatite-coating. Machined titanium surface demonstrated uniform roughness with circumferential machining marks. The hydroxyapatite coatings were advocated based on the hypothesis that this substance might improve biointegration by providing the host bone cells with a number of mineral elements involved in the metabolism of osteoblasts [42], but this biomaterial was unable to allow the stem cells to differentiate into osteoblasts without exogenous soluble factors [35].

### 4.3. AD-MSCs Resources, Isolation, Culture and Characterization

MSCs are frequently used cell types for cell-based therapeutics. As for other cell types intended for research and translational use, it is important to correctly establish the cell research, isolation methodology, culture and characterization. The frequency of MSCs in the vascular stromal fraction of adipose tissue is 2% [43], while, in bone marrow, it is 0.3% [44].

This present review demonstrated a huge discrepancy in the different origins of the AD-MSCs, as 86% derived from liposuction and 14% from bichectomy. Probably, this difference is due to the fact that the studies using mesenchymal stem cells originating from liposuction started in the beginning of the XXI century with the study of Zuk et al., 2001; the first paper using these cells, derived from buccal fat pad, was published by Farré-Guasch et al., 2010, totalizing 9 years of difference [45,46]. Furthermore, we believe that liposuction is a more common procedure than bichectomy, making this source more accessible.

Ferrin et al., 2017, described methods for isolating AD-MSCs that have been used in the context of a biobank, prepared as standard operating procedures (SOPs), ensuring traceability and reproducibility of cell production [47]. In their paper, they showed the enzymatic methodology using collagenase type I in the concentration of 0.08%. Regarding the isolation methodology, most of the articles retrieved used the enzymatic methodology, probably because obtaining cells is faster and the cell yield is better. Different types of collagenase enzymes were used for the isolation of AD-MSCs in the articles selected, but the collagenase type I was the most used in lipoaspirate and bichectomy. It was represented by a quantity close to 50% of the articles and the second most used was the collagenase type II, though it represented approximately 15% of the cases in both cell sources.

About the cell culture passages, as previously said, almost 10.8% of the reviewed articles did not give information about the cell passage. That may have happened because it is a pattern to use early passages in studies focusing on stromal cells. Hence, the authors might have thought it would not be necessary to specify the culture passage used in the experiments. It is known that, as the passage number increases, cell health proportionally decreases. Thus, a higher number of passages does not ensure consistent results for experiments, when compared with lower passages [22]. Therefore, the most indicated passages for research using stromal cells for the application in regenerative medicine are early passages (above P5) [48]. This is because, in the earlier passages, the cell culture may not be completely purified, so there may be some parental cells in the culture. Moreover, it is not indicated to use higher passages because there is a greater probability that the stromal cells suffer mutations in their genome to adapt to ex vivo conditions [49]. In our review, we understand this pattern has been followed, since almost 67% of the articles used an adequate number of passages for research using stem cells, meaning their results are more assured to be consistent. The consequences of using higher passages can be observed in one of the articles reviewed, where the research used three different passages (P2, P7 and P15) for bone reconstruction of the maxillofacial region [50]. From their article results, in one of the four samples, only P15 demonstrated a trisomy, which may demonstrate genomic instability.

MSCs are not a completely homogeneous population. Their properties can vary a lot depending on several parameters [51]. Thus, it is necessary to characterize the population used in the study in order to achieve a good result in regenerative medicine. In our analyses, as shown in Figure 9A, most articles did not mention adhesion tests, morphology and surface markers. In general, it can now be seen that the authors are more concerned about showing the differentiation tests. During the preparation of this review, questions were raised about whether these first three criteria were considered as basic or implied in the works. In the first positioning of the ISCT for MSCs, the markers analyzed by flow cytometry that must be present are: CD105, CD73 AND CD90 (≥95% of the population) and those that must be absent are: CD45, CD34, CD14 or CD11b, CD79α CD19 and HLA-DR [19]. In 2013, the ISCT, together with the International Federation for Therapeutics and Adipose Science (IFATS), issued a declaration characterizing more specifically the adipose tissue stem cells (AD-MSCs). AD-MSCs have markers in common with other stromal mesenchymal stem cells (MSCs), including CD90, CD73, CD105 and CD44 and remain negative for CD45 and CD31 [52]. Our analyses found that the authors choose some of the markers, when this analysis is present in the work, to evaluate by flow cytometry (Figure 9C). We observed that four positive markers are chosen in most studies (CD90, CD105, CD34 and CD73) and a negative one is more prevalent (CD45). Making a general observation, the articles analyzed by this review, which cited the characterization of the cells used, addressed the criteria established by ISCT. However, there were also studies that used some other markers outside these guidelines, in addition to the positive marker CD44 [52] being neglected.4.4. In vitro and in vivo assays using biomaterials and AD-MSC for regenerative medicine.

A great variety of in vitro assays was observed, which may be explained by the different focus of each experimental article; those in vitro assays described in few articles and too specific to be added to the bigger categories were grouped into the “Others” category. Those included Fourier-transform infrared spectroscopy, crystallography, X-ray diffraction assays, micro-computerized tomography, immunoblotting, rheology assays, mechanical tests, extracellular matrix assays, thermogravimetric assays, differential temperature assessments and non-metabolic assays of specific bioproducts, among others. Optical, fluorescent, scanning probe and atomic force-based microscopies [53] were pooled into a single category and, similarly, both general-purpose spectrometers and microplate readers were grouped into one.

Despite recommendations for the characterization criteria [52], a large percentage of the articles did not describe any cluster of differentiation assays in any methodology. Additionally, although most articles described some kind of microscopy technique, three articles did not describe any type of microscopy essay [54,55,56]. Some articles were vague on the description of the assays or outright only affirmed that a type of testing was performed, without elaborating further—something observed for all categories of testing. Moreover, more commonly, in many more instances, there was a detailing of the assay by linking a previous referenced article, which did not have satisfactory information about the assay itself—this was observed mainly on differentiation characterizations and, in the case of one article, on a genetic testing assay [57]. 

The results and great variability on assay types and methodologies demonstrate a lack of followed standards for the testing and characterization of stem cells in research. Those findings are in line with similar recent reviews on the characterization and testing of different stem cell types [51,58] and also with similar challenges, due to lack of standards for cell line characterization and testing [59].

The number of bone procedures is growing due to several factors, such as an aging population, obesity, traffic accidents, cancer and others. It is estimated that more than 3.5 million bone grafts are performed every year, making it the second most transplanted material in the world, after blood. Although bone tissue has a high capacity for regeneration, only 28% of patients suffering from severe open fractures can fully recover [60]. Even though our results have shown that almost 50% of the analyzed articles aimed at bone regeneration, the search for more effective treatments for bone defects should still be the goal of future research, because the numbers of clinical trials involving stem cells in bone diseases worldwide registered on the website of clinical trials are one in South America, eight in the United States, two in Europe, two in the Middle East and seven in Asia [61].

In our review, rodent models were the most used in in vivo tests and in vivo assays, probably because mice and rats are preferred when doing biomedical researches; the reason for that is their ease of preservation, cost, maintenance and small life cycle, when compared to other models [62]. Only one article [32] used New Zealand rabbits (Lagomorpha) as animal models for experimental research in the in vivo assay; this article involved a precise surgery and rabbits are anatomically bigger than mouse models, justifying that choice [63]. The use of chicken chorioallantoic membrane (CAM) as an in vivo Galliformes model draws attention, well known in the literature as a good model for viral infections, tissue grafts, drug delivery and tumor growth. There are advantages that can be attributed to this model, such as low cost, reduced incubation time, less physical space and no need for approval from the ethical committee, because it is not considered as a living animal [64]. In the three articles, the use of CAM showed good results in experiments with MSC. Of the 37 articles that used rodents as a model, 20 of them used Nude Mice, as they are immunosuppressed and have T-cell deficiency, making them a great experimentation model due to the low rejection of human stem cells derived from adipose tissue [65].

## 5. Conclusions

**Figure 12:**
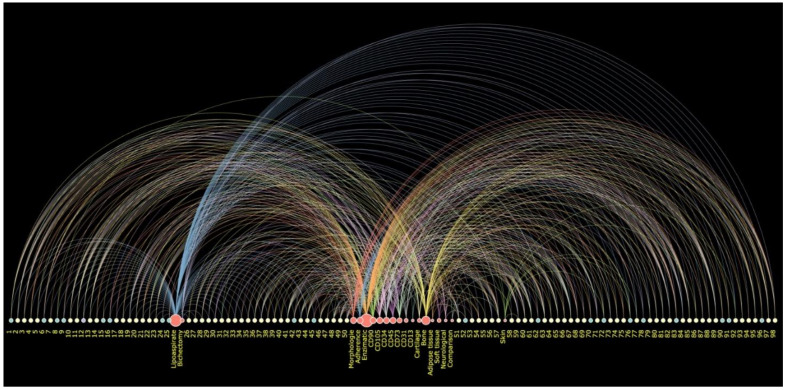
Interactive arc diagram (on, mouse hover; out) https://arc-diagram-gep.tiiny.site/ (accessed on 29 June 2021). The standard small-sized yellow/cian//lilac nodes represent each of the 98 articles (1–98 numbers on diagram). Using on-mouse hover, the yellow nodes/arcs show the articles with 50–60% positive sign from authors in answering the required protocol questions. The green nodes/arcs represent the articles with a positive sign in 70% or more questions. The lilac node/arc represents the article which contained less than 50% positive sign from questions of the protocols required according to the quality standard assessed in this review. The red nodes (different sizes) represent the origin (liposuction/bichectomy), the main characterization and differentiation analyses and, finally, the clinical application (bone, cartilage, skin, adipose tissue, soft tissue, neurological or origin cell comparison) used individually. The size of the red nodes represents the quantity of articles presenting such analyses (on, mouse hover). By hovering the mouse over each analysis (red node), the reader can visualize the breadth of the quantity of articles (quantity of lines) that was accomplished in each protocol stage.

The expansive global use of biomaterials is driven by factors of the population who are still susceptible to diseases, such as bone and cartilage, dermatologic, orthopedic and neurological conditions. The data herein analyzed were extensively used to provide a better background to the detailed protocols of translational medicine. The sum of data results elucidated the high demand for implantable gold template devices that boost research growth, being widely used in in vivo and in vitro analysis and involving mostly osteogenesis and chondrogenesis processes worldwide. The 13 types of biomaterials in these data, which are pure, double- or triple-blended biomaterials associated with AD-MSCs, also emphasized that tissue engineering fuel could be a variety of composition and a possibility for the synthetization of biomaterials. The interactive arc of the diagram represents each of the topics and breadth of the 98 articles analyzed in our review (Figure 12).

## Figures and Tables

**Table 1:** Types of in vitro assays.

Selected Articles	Number of Articles	%
Electronic Microscopy	51	52.04
Microscopy	92	93.88
ELISA	19	19.39
Immunohistochemistry	24	24.49
Immunocytochemistry	17	17.35
Spectrometry	45	45.92
Genetic testing	61	62.24
Metabolic assays	49	50.00
Viability and cytotoxicity	32	32.66
Flow cytometry	32	32.66
Others	44	44.90

## Data Availability

Data is available upon request from authors.

## References

[1] Matichescu A., Ardelean L.C., Rusu L.-C., Craciun D., Bratu E.A., Babucea M., Leretter M. (2020). Advanced Biomaterials and Techniques for Oral Tissue Engineering and Regeneration—A Review. Materials.

[2] Raghavendra G.M., Varaprasad K., Jayaramudu T. (2015). Biomaterials. Nanotechnology Applications for Tissue Engineering.

[3] Solanki A., Kim J.D., Lee K.-B. (2008). Nanotechnology for regenerative medicine: Nanomaterials for stem cell imaging. Nanomedicine.

[4] Pryjmaková J., Kaimlová M., Hubáček T., Švorčík V., Siegel J. (2020). Nanostructured Materials for Artificial Tissue Replacements. Int. J. Mol. Sci..

[5] Zhang L., Webster T.J. (2009). Nanotechnology and nanomaterials: Promises for improved tissue regeneration. Nano Today.

[6] Verma S., Domb A.J., Kumar N. (2011). Nanomaterials for regenerative medicine. Nanomedicine.

[7] Viswanathan S., Shi Y., Galipeau J., Krampera M., Leblanc K., Martin I., Nolta J., Phinney D.G., Sensebe L. (2019). Mesenchymal stem versus stromal cells: International Society for Cell & Gene Therapy (ISCT(R)) Mesenchymal Stromal Cell committee position statement on nomenclature. Cytotherapy.

[8] Harasymiak-Krzyżanowska I., Niedojadło A., Karwat J., Kotuła L., Gil-Kulik P., Sawiuk M., Kocki J. (2013). Adipose tissue-derived stem cells show considerable promise for regenerative medicine applications. Cell. Mol. Biol. Lett..

[9] Afra S., Matin M.M. (2020). Potential of mesenchymal stem cells for bioengineered blood vessels in comparison with other eligible cell sources. Cell Tissue Res..

[10] Hentze H., Soong P.L., Wang S.T., Phillips B.W., Putti T.C., Dunn N.R. (2009). Teratoma formation by human embryonic stem cells: Evaluation of essential parameters for future safety studies. Stem Cell Res..

[11] Brown C., McKee C., Bakshi S., Walker K., Hakman E., Halassy S., Svinarich D., Dodds R., Govind C.K., Chaudhry G.R. (2019). Mesenchymal stem cells: Cell therapy and regeneration potential. J. Tissue Eng. Regen. Med..

[12] Ullah I., Subbarao R.B., Rho G.J. (2015). Human mesenchymal stem cells—Current trends and future prospective. Biosci. Rep..

[13] Wright A., Arthaud-Day M.L., Weiss M.L. (2021). Therapeutic Use of Mesenchymal Stromal Cells: The Need for Inclusive Characterization Guidelines to Accommodate All Tissue Sources and Species. Front. Cell Dev. Biol..

[14] Salehi-Nik N., Rad M.R., Kheiri L., Nazeman P., Nadjmi N., Khojasteh A. (2017). Buccal Fat Pad as a Potential Source of Stem Cells for Bone Regeneration: A Literature Review. Stem Cells Int..

[15] Cherian D.S., Bhuvan T., Meagher L., Heng T.S.P. (2020). Biological Considerations in Scaling Up Therapeutic Cell Manufacturing. Front. Pharmacol..

[16] Zhang J., Liu Y., Chen Y., Yuan L., Liu H., Wang J., Liu Q., Zhang Y. (2020). Adipose-Derived Stem Cells: Current Applications and Future Directions in the Regeneration of Multiple Tissues. Stem Cells Int..

[17] Moher D., Liberati A., Tetzlaff J., Altman D.G., The PRISMA Group (2009). Preferred Reporting Items for Systematic Reviews and Meta-Analyses: The PRISMA Statement. PLoS Med..

[18] Higgins J.P.T., Thomas J., Chandler J., Cumpston M., Li T., Page M.J., Welch V.A. (2019). Cochrane Handbook for Systematic Reviews of Interventions.

[19] Dominici M., Le Blanc K., Mueller I., Slaper-Cortenbach I., Marini F., Krause D., Deans R., Keating A., Prockop D., Horwitz E. (2006). Minimal criteria for defining multipotent mesenchymal stromal cells. The International Society for Cellular Therapy position statement. Cytotherapy.

[20] Pires A.L.R., Bierhalz A.C.K., Moraes A.M. (2015). Biomateriais: Tipos, Aplicações e Mercado. Quim. Nova.

[21] Buddy D., Ratner A., Hoffman S., Frederick J., Schoen J., Lemons E. (2013). Biomaterials Science.

[22] Kwist K., Bridges W.C., Burg K.J.L. (2015). The effect of cell passage number on osteogenic and adipogenic characteristics of D1 cells. Cytotechnology.

[23] Yu X., Tang X., Gohil S.V., Laurencin C.T. (2015). Biomaterials for Bone Regenerative Engineering. Adv. Health Mater..

[24] Qi C., Yan X., Huang C., Melerzanov A., Du Y. (2015). Biomaterials as carrier, barrier and reactor for cell-based regenerative medicine. Protein Cell.

[25] Singh M., Kundu S., Motiani R., Sreekanth V., Motiani R.K., Sengupta S., Srivastava A., Bajaj A. (2014). Injectable small molecule hydrogel as a potential nanocarrier for localized and sustained in vivo delivery of doxorubicin. Nanoscale.

[26] Perez-Estenaga I., Prosper F., Pelacho B. (2018). Allogeneic Mesenchymal Stem Cells and Biomaterials: The Perfect Match for Cardiac Repair?. Int. J. Mol. Sci..

[27] Hung B.P., Naved B.A., Nyberg E.L., Dias M., Holmes C., Elisseeff J.H., Dorafshar A., Grayson W.L. (2016). Three-Dimensional Printing of Bone Extracellular Matrix for Craniofacial Regeneration. ACS Biomater. Sci. Eng..

[28] Jeong S.I., Kim S.H., Kim Y.H., Jung Y., Kwon J.H., Kim B.-S., Lee Y.M. (2004). Manufacture of elastic biodegradable PLCL scaffolds for mechano-active vascular tissue engineering. J. Biomater. Sci. Polym. Ed..

[29] Huri P.Y., Ozilgen B.A., Hutton D.L., Grayson W.L. (2014). Scaffold pore size modulates in vitro osteogenesis of human adipose-derived stem/stromal cells. Biomed. Mater..

[30] Cardoso G.B., Chacon E., Chacon P.G., Bordeaux-Rego P., Duarte A.S., Saad S.T.O., Zavaglia C.A., Cunha M.R. (2017). Fatty acid is a potential agent for bone tissue induction: In vitro and in vivo approach. Exp. Biol. Med..

[31] Zhang K., Song L., Wang J., Yan S., Li G., Cui L., Yin J. (2017). Strategy for constructing vascularized adipose units in poly(l-glutamic acid) hydrogel porous scaffold through inducing in-situ formation of ASCs spheroids. Acta Biomater..

[32] Denost Q., Adam J.-P., Pontallier A., Montembault A., Bareille R., Siadous R., Delmond S., Rullier E., David L., Bordenave L. (2015). Colorectal tissue engineering: A comparative study between porcine small intestinal submucosa (SIS) and chitosan hydrogel patches. Surgery.

[33] Ribeiro V., Correia J.S., Nascimento A.I., Morais A.D.S., Marques A., Ribeiro A.S., Silva C., Bonifácio G., Sousa R., Oliveira J.M. (2017). Silk-based anisotropical 3D biotextiles for bone regeneration. Biomaterials.

[34] Bellas E., Panilaitis B.J., Glettig D.L., Kirker-Head C.A., Yoo J.J., Marra K., Rubin J.P., Kaplan D.L. (2013). Sustained volume retention in vivo with adipocyte and lipoaspirate seeded silk scaffolds. Biomaterials.

[35] Park J.-B., Kim Y.S., Lee G., Yun B.G., Kim C.-H. (2013). The effect of surface treatment of titanium with sand-blasting/acid-etching or hydroxyapatite-coating and application of bone morphogenetic protein-2 on attachment, proliferation, and differentiation of stem cells derived from buccal fat pad. Tissue Eng. Regen. Med..

[36] Bastami F., Paknejad Z., Jafari M., Salehi M., Rad M.R., Khojasteh A. (2017). Fabrication of a three-dimensional β-tricalcium-phosphate/gelatin containing chitosan-based nanoparticles for sustained release of bone morphogenetic protein-2: Implication for bone tissue engineering. Mater. Sci. Eng. C.

[37] Choi J.S., Yang H.-J., Kim B.S., Kim J.D., Lee S.H., Lee E.K., Park K., Cho Y.W., Lee H.Y. (2010). Fabrication of Porous Extracellular Matrix Scaffolds from Human Adipose Tissue. Tissue Eng. Part C: Methods.

[38] Chattopadhyay A., Galvez M.G., Bachmann M., Legrand A., McGoldrick R., Lovell A., Jacobs M., Crowe C., Umansky E., Chang J. (2016). Tendon Regeneration with Tendon Hydrogel–Based Cell Delivery: A Comparison of Fibroblasts and Adipose-Derived Stem Cells. Plast. Reconstr. Surg..

[39] Eke G., Mangir N., Hasirci N., MacNeil S., Hasirci V. (2017). Development of a UV crosslinked biodegradable hydrogel containing adipose derived stem cells to promote vascularization for skin wounds and tissue engineering. Biomaterials.

[40] Tatrai P., Sági B., Szigeti A., Szepesi Á., Szabó I., Bosze S., Kristóf Z., Markó K., Szakacs G., Urbán I. (2012). A novel cyclic RGD-containing peptide polymer improves serum-free adhesion of adipose tissue-derived mesenchymal stem cells to bone implant surfaces. J. Mater. Sci. Mater. Med..

[41] Razavi S., SeyedEbrahimi R., Jahromi M. (2019). Biodelivery of nerve growth factor and gold nanoparticles encapsulated in chitosan nanoparticles for schwann-like cells differentiation of human adipose-derived stem cells. Biochem. Biophys. Res. Commun..

[42] Doglioli P., Scortecci G., Falatouni M. (2001). A novel spectrofluorometric technique for specific biocompatibility testing of implantable materials by cell culture. Report on use for multiparameter analysis of human osteoblasts cultured on commercially pure titanium and hydroxyapatite. Cytotechnology.

[43] Zuk P.A., Zhu M., Ashjian P., De Ugarte D.A., Huang J.I., Mizuno H., Alfonso Z.C., Fraser J.K., Benhaim P., Hedrick M.H. (2002). Human Adipose Tissue Is a Source of Multipotent Stem Cells. Mol. Biol. Cell.

[44] Sarugaser R., Lickorish D., Baksh D., Hosseini M.M., Davies J.E. (2005). Human Umbilical Cord Perivascular (HUCPV) Cells: A Source of Mesenchymal Progenitors. Stem Cells.

[45] Zuk P.A., Zhu M., Mizuno H., Huang J., Futrell J.W., Katz A.J., Benhaim P., Lorenz H.P., Hedrick M.H. (2001). Multilineage Cells from Human Adipose Tissue: Implications for Cell-Based Therapies. Tissue Eng..

[46] Farré-Guasch E., Martí-Pagès C., Hernández-Alfaro F., Klein-Nulend J., Casals N. (2010). Buccal Fat Pad, an Oral Access Source of Human Adipose Stem Cells with Potential for Osteochondral Tissue Engineering: An In Vitro Study. Tissue Eng. Part C Methods.

[47] Ferrin I., Beloqui I., Zabaleta L., Salcedo J.M., Trigueros C., Martin A.G., Crook J., Ludwig T. (2017). Isolation, Culture, and Expansion of Mesenchymal Stem Cells. Stem Cell Banking. Methods in Molecular Biology.

[48] Jiang T., Xu G., Wang Q., Yang L., Zheng L., Zhao J., Zhang X. (2017). In vitro expansion impaired the stemness of early passage mesenchymal stem cells for treatment of cartilage defects. Cell Death Dis..

[49] Pamies D., Bal-Price A., Simeonov A., Tagle D., Allen D., Gerhold D., Yin D., Pistollato F., Inutsuka T., Sullivan K. (2017). Good Cell Culture Practice for Stem Cells and Stem-Cell-Derived Models. Altex.

[50] Bellotti C., Stanco D., Ragazzini S., Romagnoli L., Martella E., Lazzati S., Marchetti C., Donati D.M., Lucarelli E. (2013). Analysis of the Karyotype of Expanded Human Adipose-Derived Stem Cells for Bone Reconstruction of the Maxillo-Facial Region. Int. J. Immunopathol. Pharmacol..

[51] Mushahary D., Spittler A., Kasper C., Weber V., Charwat V. (2018). Isolation, cultivation, and characterization of human mesenchymal stem cells. Cytom. Part A.

[52] Bourin P., Bunnell B., Casteilla L., Dominici M., Katz A.J., March K.L., Redl H., Rubin J.P., Yoshimura K., Gimble J.M. (2013). Stromal cells from the adipose tissue-derived stromal vascular fraction and culture expanded adipose tissue-derived stromal/stem cells: A joint statement of the International Federation for Adipose Therapeutics and Science (IFATS) and the International Society for Cellular Therapy (ISCT). Cytotherapy.

[53] Müller D.J., Dufrêne Y. (2011). Atomic force microscopy: A nanoscopic window on the cell surface. Trends Cell Biol..

[54] Heo S.C., Shin W.C., Lee M.J., Kim B.R., Jang I.H., Choi E.-J., Lee J.S., Kim J.H. (2015). Periostin Accelerates Bone Healing Mediated by Human Mesenchymal Stem Cell-Embedded Hydroxyapatite/Tricalcium Phosphate Scaffold. PLoS ONE.

[55] Grotenhuis N., De Witte S.F., Van Osch G.J., Bayon Y., Lange J.F., Bastiaansen-Jenniskens Y.M. (2016). Biomaterials Influence Macrophage–Mesenchymal Stem Cell Interaction In Vitro. Tissue Eng. Part A.

[56] Douglas T., Vandrovcová M., Kročilová N., Keppler J.K., Zárubová J., Skirtach A.G., Bacakova L. (2018). Application of whey protein isolate in bone regeneration: Effects on growth and osteogenic differentiation of bone-forming cells. J. Dairy Sci..

[57] Ma J., Yang F., Both S.K., Prins H.-J., Helder M.N., Pan J., Cui F.-Z., Jansen J.A., Beucken J.J.V.D. (2014). Bone forming capacity of cell- and growth factor-based constructs at different ectopic implantation sites. J. Biomed. Mater. Res. Part A.

[58] Samsonraj R.M., Raghunath M., Nurcombe V., Hui J.H., Van Wijnen A.J., Cool S.M. (2017). Concise Review: Multifaceted Characterization of Human Mesenchymal Stem Cells for Use in Regenerative Medicine. Stem Cells Transl. Med..

[59] Loring J.F., Rao M.S. (2006). Establishing Standards for the Characterization of Human Embryonic Stem Cell Lines. Stem Cells.

[60] Henkel J., Woodruff M., Epari D., Steck R., Glatt V., Dickinson I.C., Choong P., Schuetz M.A., Hutmacher D.W. (2013). Bone Regeneration Based on Tissue Engineering Conceptions—A 21st Century Perspective. Bone Res..

[61] Clinical Trials Clinical Trials.Gov. https://clinicaltrials.gov/ct2/results/map?term=adipose+stem+cells&cond=bone&map.

[62] Bryda E.C. (2013). The Mighty Mouse: The Impact of Rodents on Advances in Biomedical Research. Mo. Med..

[63] Zolocinska A., Siennicka K., Debski T., Gut G., Mazur S., Gajewska M., Kaminski A., Pojda Z. (2020). Comparison of mouse, rat and rabbit models for adipose—Derived stem cells (ASC) research. Curr. Res. Transl. Med..

[64] Ribatti D. (2016). The chick embryo chorioallantoic membrane (CAM). A multifaceted experimental model. Mech. Dev..

[65] Moticka E.J. (2016). The Thymus in Lymphocyte Maturation. A Historical Perspective on Evidence-Based Immunology.

